# Comparison of Rain-Fast Bait Stations Versus Foliar Bait Sprays for Control of Oriental Fruit Fly, *Bactrocera dorsalis*, in Papaya Orchards in Hawaii

**DOI:** 10.1673/031.010.14117

**Published:** 2010-09-20

**Authors:** Jaime C. Piñero, Ronald F. L. Mau, Roger I. Vargas

**Affiliations:** ^1^ University of Hawaii at Manoa, College of Tropical Agriculture and Human Resources, 3050 Maile Way, Honolulu, HI 96822; ^2^ U.S. Pacific Basin Agricultural Research Center, USDA-ARS, P.O. Box 4459, Hilo, HI 96720; ^3^ Current address: Lincoln University of Missouri, Cooperative Research and Extension, Allen Hall 212, Jefferson City, Missouri, 65101

**Keywords:** attract-and-kill, control, IPM, papaya leaf mimic, sanitation, Tephritidae

## Abstract

Bait stations represent an environmentally friendly attract-and-kill approach to fruit fly population suppression. Recently a novel, visually attractive, rain-fast bait station was developed in Hawaii for potential use against multiple species of pestiferous fruit flies. Here, we compared the efficacy of GF-120 NF Naturalyte Fruit Fly Bait applied either as foliar sprays or onto bait stations in reducing female oriental fruit fly, *Bactrocera dorsalis* (Hendel) (Diptera: Tephritidae), population density and level of fruit infestation in commercial papaya orchards in Hawaii. Trapping and infestation data were used as indicators of the effectiveness of the two bait application methods. For the first 10 weeks of the study, captures of female *B. dorsalis* in monitoring traps were significantly greater in control plots than in plots treated with foliar sprays or bait stations. Six weeks after the first bait spray, incidence of infestation (i.e. number of fruit with one or more *B. dorsalis* larvae) of quarter to half-ripe papaya fruit was reduced by 71.4% and 63.1% for plots with bait stations and foliar sprays, respectively, as compared to control plots. Twelve weeks after first spray, incidence of infestation was reduced by only 54.5% and 45.4% for plots with bait stations and foliar sprays, respectively, as compared to control plots. About 42% less GF-120 was used in orchard plots with bait stations compared to those subject to foliar sprays. The impact of field sanitation on the outcome is also discussed. The results indicate that bait stations can provide a simple, efficient, and economical method of applying insecticidal baits to control fruit flies and a safer alternative to foliar sprays.

## Introduction

For decades, management of pestiferous fruit flies (Diptera: Tephritidae) in various areas of the world relied heavily upon the application of protein baits mixed with highly toxic organophosphate insecticides such as malathion (Steiner et al. 1961; Roessler 1989; Vargas et al. 2005). More recently, improved behavioral approaches to pest management such as attract-and-kill systems that use reduced-risk insecticides have proven to be an excellent alternative to the conventional application of broad spectrum insecticides (Shelton & Badenes-Perez 2006; Cook et al. 2007).

For nearly a decade (1999–2008), the Hawaii Area-Wide Fruit Fly Pest Management (HAWPM) program developed biologicallybased approaches for area-wide suppression of economically important species of invasive fruit flies such as the Mediterranean fruit fly, *Ceratitis capitata;* oriental fruit fly, *Bactrocera dorsalis* (Coquillett) (Hendel); and melon fly, *B. Cucurbitae* throughout selected agricultural areas of Hawaii (Mau et al. 2007; Vargas et al. 2008, 2010). Since its conception in 1999, the HAWPM program effectively integrated key fruit fly control tactics such as sanitation, male annihilation through mass trapping, and female-targeted bait sprays using first GF-120 Fruit Fly Bait and later the most recent organic formulation GF-120 NF Naturalyte Fruit Fly Bait (Dow Agrosciences, www.dowagro.com). This spinosadcontaining bait has proven effective against *B. cucurbitae* (Jang et al. 2008), *C. capitata* (Vargas et al. 2010), and *B. dorsalis* (Piñero et al. 2009a) and consequently has become the primary tool for area-wide suppression of tephritid fruit flies in the Hawaiian Islands (Vargas et al. 2008).

Factors such as rainfall (Piñero et al. 2009a) and phytotoxicity (DeLury et al. 2009) may influence the efficacy or utility of foliar applications of insecticidal baits. In a previous study (Piñero et al. 2009a) rainfall that fell during or shortly after foliar applications of GF-120 in commercial papaya, *Carica papaya* L. (Brassicales: Caricaceae), orchards reduced bait effectiveness. In an attempt to overcome this problem, a visually attractive rain-fast bait station was developed (Piñero et al. 2009b). The bait station was termed a Papaya Leaf Mimic (PLM) because it represents a supernormal visual stimulus of papaya foliage and serves as an attract-and-kill system to which insecticidal baits can be applied. Intensive research has demonstrated that PLMs not only protect GF-120 against rainfall but also enhance the behavioral response of adult fruit flies to this bait and extend its attractiveness for at least one week (Piñero et al. 2009b). Furthermore, the application of insecticidal baits onto PLMs circumvents the phytotoxicity caused by this bait on some crops (DeLury et al. 2009) and minimizes degradation of spinosad by photolysis (Mangan et al. 2006).

For PLMs to be considered by fruit and vegetable growers as a viable alternative to foliar bait sprays, they should be costcompetitive and show good performance in commercial orchards. The goal of this largescale study was to compare the efficacy of GF-120 NF Naturalyte Fruit Fly Bait when applied either as foliar sprays or in PLMs in reducing the abundance of female *B. dorsalis* and the level of fruit infestation in papaya orchards in Hawaii.

## Materials and Methods

### Study Site

**Figure 1.**
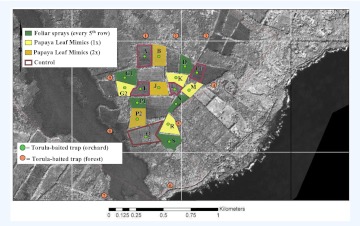
The experimental area in Puna, Hawaii. Except for control plots (n = 5), all plots received GF-120 NF Naturalyte Fruit Fly Bait applied either to papaya tree foliage using a 10% solution, or onto PLMs (1x = bait applied once a week; 2x = bait applied twice a week) using a 20% solution of GF-120. Only plots (n = 17) with a green circle (denoting a torula-baited McPhail trap deployed at the center of each experimental plot) were used for data collection. Adjacent plots were also sprayed to minimize buildups. High quality figures are available online.

This investigation was conducted in a papaya-growing area located in the Puna area of Hawaii Island. The area in production comprised about 130 ha and had been utilized in a previous evaluation of various spray patterns of GF-120 against *B. dorsalis* (Piñero et al. 2009a). For the present study, 17 orchard plots (mean plot area ± SEM: 2.02 ± 0.24 ha) were utilized (Figure 1). The predominant papaya cultivars planted in the experimental plots were ‘Rainbow’ (ca. 60%) and ‘Sunrise’ (ca.30%). Each grower managed diseases by means of weekly preventive applications of fungicides such as Manzate and Dithane (Mancozeb) to both foliage (against black spot fungus, *Asperisporium caricae*) and fruit (against phytophthora blight, *Phytophthora parasitica*). No insecticides were applied during the study period.

### Papaya Leaf Mimics

**Figure 2.**
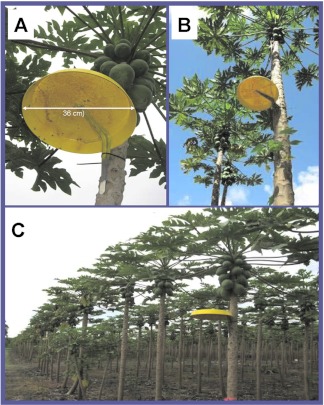
(A) A yellow Papaya Leaf Mimic (PLM) showing adult *Bactrocera dorsalis* and B. *cucurbitae* feeding on GF-120 NF, (B) view of a PLM attached to a papaya tree trunk, and (C) PLM deployment on perimeter-row trees in a papaya orchard. High quality figures are available online.

Bait stations were constructed as described in Piñero et al. (2009b). In short, they consisted of inverted plant pot saucers (36 cm outer diameter; 5 cm deep) to which a metal shelf bracket (20.3 × 25.4 cm) was attached with screws and glue (Gorilla Glue, Cincinnati, Ohio, USA) for easy fastening to *C. papaya* tree trunks using zip ties (Figure 2). The interior area of each saucer was heavily scraped in a circular fashion using a wirewheel brush to increase adherence of the bait. Each bait station was then painted yellow using spray paint (Krylon Products Group, www.kpg-industrial.com). For further details see Piñero et al. (2009b).

### Bait Spray Treatments

Four treatments were compared using a completely randomized design: (1) GF-120 NF Naturalyte Fruit Fly Bait (hereafter referred to as GF-120) (Dow AgroSciences LLC) applied weekly to the foliage of papaya trees (n = 5 plots), (2) GF-120 applied weekly to the interior surface of PLMs (n = 4), (3) as in (2) but with bait applied to PLMs twice a week (Tuesdays and Fridays) (n = 3), and (4) control plots that did not receive any bait application (n = 5). Bait applications started on 18 March and continued weekly for 12 weeks until 10 June 2008.

Applications of GF-120 (either to papaya tree foliage or onto PLMs) were conducted by a team of four persons in the morning hours (08:00 – 11:00). Foliar sprays were applied with backpack sprayers (capacity 12 1) mounted on all-terrain vehicles using a pressure of about 20 psi. Foliar sprays were applied to all trees in every fifth row. Each sprayed tree received about 10 ml of a 10% bait solution. This spray pattern, dilution rate, and volume per tree had proven effective in a previous field study when conducted in association with thorough fruit sanitation practices (Piñero et al. 2009a).

PLMs were deployed every 20 m along the perimeter of each plot and also in interior trees (every 5^th^ row) at an average density of 30 units per ha. In the absence of published information on the range of attraction of adult *Bactrocera* spp. to visual traps and/or to olfactory (protein-based) stimuli, the distance between PLMs was somewhat arbitrary, but took into consideration recommended distances among odor-baited traps in Massachusetts apple orchards for control of the apple maggot fly, *Rhagoletis pomonella* (e.g. Bostanian & Racette 2001; Prokopy et al. 2003, 2005). Each PLM received ca. 20 ml of a 20% bait solution either once (for treatment 2) or twice (for treatment 3) per week. Bait was applied with hand-held Delta pressure sprayers (capacity 1.4 1) (Delta Industries, www.deltasprayers.com). The 20% dilution rate is as attractive as the label-recommended dilution rate of 40% over a 7-day period (Piñero et al. 2009b). On 5 May (i.e. 8 weeks after the first bait spray), all PLMs were removed and washed thoroughly with water in the field to eliminate incipient mold growth (detected in about 20% of the PLMs). PLMs were re-deployed and re-sprayed on 6 May.

It is important to highlight that, in a previous evaluation (Piñero et al. 2009a), applications of GF-120 targeted both the foliage of papaya trees (using the 10% solution) as well as border plants adjacent to treated areas (using a 40% solution) due to very high *B. dorsalis* pressure. This spray pattern resulted in substantially more (ca. 80% more) bait being sprayed every week to border areas than to papaya tree foliage. In the present study, border areas were not sprayed due to lack of perceived cost-effectiveness because *B. dorsalis* populations were comparatively low at the onset of the study.

### Monitoring Traps.

The relative abundance of female *B. dorsalis* in each of the 17 experimental plots was quantified on a weekly basis from 9 January until 18 June 2008 using McPhail-type traps baited with 300 ml of a torula yeast (ERA International, Ltd.) solution (1 pellet per 100 ml of water). Each of the 17 experimental plots received one monitoring trap, deployed at the plot center. Trap capture data collected during the actual bait spray periods (18 March – 10 June 2008) were used for the determination of the effectiveness of the two application methods. Nine torula-baited traps were deployed in forested areas adjacent to the experimental plots (Figure 1) to obtain an estimate of the relative abundance of *B. dorsalis* outside the study area, i.e. potential immigrants). Forested areas contained large patches of strawberry guava (*Psidium cattleianum* Sabine) and common guava (*P. guajava* L.) (Myrtaceae) both of which are preferred host plants of *B. dorsalis* in Hawaii, and major sources of flies that move into agricultural areas (Vargas et al. 1989, 1990).

All captured flies were transported to the laboratory in plastic bags for identification and sexing, but only female numbers are reported herein. Numbers of male *B. dorsalis* were suppressed by means of 540 bucket traps (for a description see Vargas et al. 2003) that were baited with the highly attractive male-specific lure methyl eugenol (ME) (Metcalf and Metcalf 1992). Traps were deployed at a density of 10–12 traps per ha in a grid that covered the entire experimental area and extended 200 m inside the forested area located on the north side. Consequently, ME traps were not used as a treatment factor in this study.

### Fruit Infestation

Papayas were sampled from each experimental plot to provide an evaluation of the effectiveness of the bait sprays. Infestation data were collected six weeks (on 5 May) and 12 weeks (on 16 June) after initiation of the bait sprays. For each of the two sampling dates and for each of the 17 experimental plots, 10 quarter-ripe and 10 half-ripe fruits were picked from randomly-selected (perimeter- and interior-row) trees and transported to the University of Hawaii Experiment Station at Waiakea, HI. Fruit ripeness was characterized using the qualitative descriptions reported in Liquido et al. (1989). Each sampled fruit was individually labeled with information on plot, weight, and degree of ripeness and placed in a 4-liter bucket with sand as pupation substrate. Sand was sieved twice, two and four weeks later, and all pupae recovered were placed inside plastic cups with approx. 2 cm of moist sand until adult emergence.

### Field sanitation

Papaya growers were invited to cooperate by collecting and bagging all abscised/ unharvested papayas at least once a week. Some growers were unable to practice proper sanitation, and consequently sanitation data were collected from each experimental plot to determine the influence of this cultural practice on the outcome. Sanitation data was taken from each of the 17 experimental plots on 2 May and 15 June (i.e. 1–3 days before conducting each of the two fruit samplings). For each plot, the level of field sanitation was quantified by recording the numbers of fallen fruit (harvestable size) in a sample of 10% of the rows (Piñero et al. 2009a). Sampled rows were spaced equidistantly and always included the perimeter rows. These data were used to assess the effectiveness of grower field sanitation practices in each of the 17 plots and to correlate sanitation practices with trapping and infestation data.

### Weather Data

Weather data was recorded and averaged on an hourly basis by HOBO weather loggers (Onset Computer Corporation, www.onsetcomp.com) located in plot D.

### Statistical Analyses

**Figure 3.**
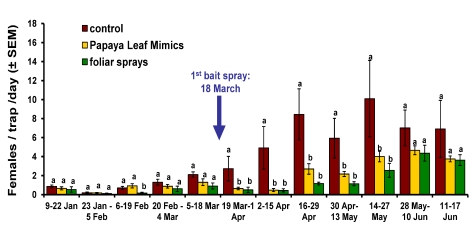
Captures (females/trap/day ± SEM) of *Bactrocera dorsalis* in 17 monitoring traps deployed in orchard plots according to bait treatment for each of five pre-bait treatment periods and for each of seven post-bait treatment periods. Columns headed by the same letter are not significantly different according to ANOVA and Fisher-protected LSD tests at α = 0.05. **For pre-bait treatment:** 9–22 January: *F*_2, 25_ = 1.01, P = 0.378; 23 January – 5 February: *F*_2, 31_ = 0.47, *P* = 0.63; 6–19 February: *F*_2, 31_ = 5.19, *P* = 0.01; 20 February – 4 March: *F*_2, 31_ = 2.15, *P* = 0.13; 5 – 18 March: *F*_2, 31_ = 1.30, *P* = 0.29. **For post-bait treatment:** 19 March – 1 April: *F*_2, 29_ = 3.49, *P* = 0.04; 2–15 April: *F*_2, 14_ = 5.22, P = 0.02; 16–29 April: *F*_2, 31_ = 8.06, *P* < 0.01; 30 April – 13 May: *F*_2, 31_ = 5.82, *P* < 0.01; 14–27 May: *F*_2, 29_ = 4.91, *P* = 0.01; 28 May – 10 June: *F*_2, 31_ = 0.85, *P* = 0.44; 11–17 June: *F*_2, 14_ = 1.06, *P* = 0.37. High quality figures are available online.

**Figure 4.**
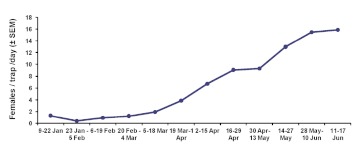
Captures (females/trap/day ± SEM) of *Bactrocera dorsalis* in nine torula-baited traps deployed in forested areas adjacent to the experimental plots for a 23-week period. Data show 2-week captures. High quality figures are available online.

A preliminary analysis revealed no significant differences in weekly captures of females (expressed as numbers of females/trap/day) or in levels of fruit infestation by *B. dorsalis* in plots with PLMs sprayed once a week (treatment 2) versus twice a week (treatment 3). Therefore, trap capture and fruit infestation data were combined into a single PLM treatment (with a resulting n = 7). Weekly captures of female *B. dorsalis* in monitoring traps were combined into two-week periods (five before and six after initiation of the bait sprays) and one final week period. Data for each trapping period were compared among the three resulting bait treatments (including pre-treatment plots) using one-way ANOVA on transformed data (sqrt [x + 0.5]) whenever needed to homogenize variances. Infestation data were analyzed for all fruits (i.e. quarter + half ripe) sampled. For each of the two fruit samplings, incidence of infestation (data expressed as proportions of fruit that yielded at least one *B. dorsalis* pupa) was compared among the three treatments using one-way ANOVA after arcsin transformation. Field sanitation data (expressed as the mean number of ground fruit recorded per plot per row) were compared among the three treatments using the non-parametric Kruskal-Wallis test. A non-parametric Mann-Whitney test was used to compare field sanitation levels between the two assessment dates for all treatments combined. In addition, Pearson's product-moment correlation (Pearson 1896) was used to quantify, for each of the three treatments and for each sampling date, the relationship between the numbers of ground fruit per row per plot and (1) the numbers of females trapped in the same plots and (2) incidence of fruit infestation by *B. dorsalis.* Wherever appropriate, Fisher-Protected LSD tests were used to separate means. Figures 3 and 4 and Table 1 show untransformed data. All statistical analyses were conducted using the software Statistica (StatSoft 2001).

## Results

### Fruit Fly Trapping

Before initiation of the bait sprays there were no significant differences in the numbers of female *B. dorsalis* captured in monitoring traps except for the period of 6 February - 19 February. During this period significantly more females were trapped in plots that would subsequently be assigned to control and PLM treatments, than in plots that would be assigned to foliar sprays (Figure 3). For each of the first five trapping periods (i.e. 10 weeks) that followed the first bait spray (on 18 March), significantly more females were captured by monitoring traps in control plots than in sprayed plots. No significant differences were detected between the two spray methods during these five periods (Figure 3) in despite of the substantially reduced (∼ 42%) amount of bait used weekly in PLM-treated plots compared to plots treated with foliar sprays. For the last two spray periods (28 May – 10 June and 11 June – 17 June), no significant differences in female captures were detected among treatments.

**Table 1.**
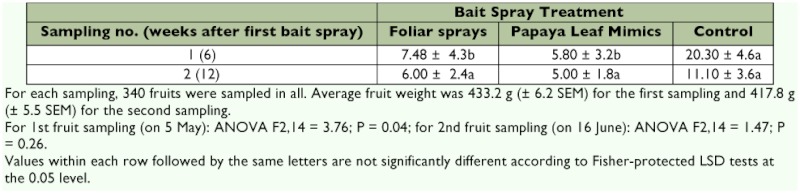
Effect of bait spray treatment on incidence of infestation of quarter-to-half ripe fruit (mean % ± SEM) by *B. dorsalis* on two sampling dates.

Captures of *B. dorsalis* females in forest traps were initially low in January with numbers gradually increasing during February and March, and sharply increasing in early April (Figure 4). The density of females recorded by the time of the first fruit sampling (9.3 females/trap/day for the period between 30 April to 13 May) was nearly half the number trapped by the time of the second fruit sampling (15.8 females/trap/day for 11 June – 17 June). The overall profile of captures in the experimental plots closely matched the seasonal occurrence of *B. dorsalis* outside the study area (Figure 4).

### Field Sanitation

**Figure 5.**
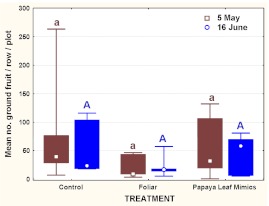
For each of two quantitative assessments of levels of field sanitation, median number of fruit per row per plot (box: 25%, 75%; whisker: Min, Max) according to bait treatment. For each assessment date, boxes headed by the same letter are not significantly different according to a Kruskal-Wallis test at α = 0.05. High quality figures are available online.

There was no significant difference in the median number of fallen fruits quantified per plot per row among the tree bait spray treatments in either assessment (KruskalWallis H = 1.37, p = 0.504 and H = 3.84, p = 0.146 for the 2 May and 15 June assessments) (Figure 5). A Mann-Whitney test revealed no significant differences (U = 133; Z = 0.40; p = 0.692) in overall field sanitation levels between the two assessment dates. Median values (25–75 quartiles) were 32 (9 – 76.5) for 2 May and 22.7 (16.3 – 63.8) for 15 June.

### Fruit Infestation

Weekly applications of GF-120, either in the form of foliar sprays or applied to PLMs, resulted in a significant reduction in the proportion of quarter to half ripe fruit infested by *B. dorsalis* compared to control plots on the first sampling date (5 May), but not on the second sampling date (16 June) (Table 1).

### Relationships among Plot Sanitation, Female Trap Captures and Fruit Infestation

For control plots, the numbers of female *B. dorsalis* captured in monitoring traps were positively correlated (r = 0.97, p = 0.008) with the numbers of unharvested, abscised fruit at the time of the first fruit sampling, but by the second sampling this relationship was nonsignificant (r = 0.67, p = 0.217). Incidence of fruit infestation in the unsprayed plots was positively correlated with numbers of fallen fruits for each of the two fruit samplings (r = 0.99, p < 0.001 and r = 0.88, p = 0.05, for the 5 May and 16 June samplings, respectively).

For plots subject to foliar sprays, the number of female *B. dorsalis* captured in monitoring traps was independent of field sanitation levels in both sampling dates (r = 0.15, p = 0.809 and r = 0.61, p = 0.271, for first and second fruit samplings, respectively). For the first sampling, there was no relationship between incidence of infestation and sanitation level (r = 0.15, p = 0.809) but for the second fruit sampling, incidence of infestation was positively correlated with numbers of fallen fruit (r = 0.89, p = 0.046).

For PLM-treated plots, no relationships between numbers of female *B. dorsalis* trapped and levels of field sanitation were noted on either of the two sampling dates (r = 0.50, p = 0.257 and r = 0.17, p = 0.708, for first and second samplings, respectively). Incidence of infestation was positively correlated with numbers of fallen fruit for the first (r = 0.83, p = 0.022), but not the second (r = 0.12, p = 0.789), fruit sampling.

**Table 2.**
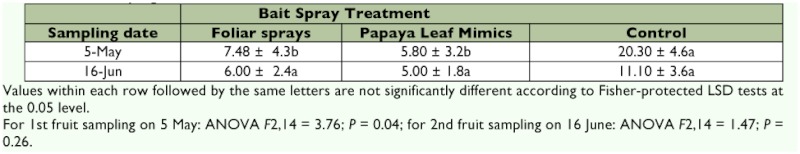
Effect of bait spray treatment on incidence of fruit (quarter-to-half ripe) infestation level (mean % ± SEM) by B. dorsalis on two sampling dates.

### Weather conditions

Mean daily air temperatures during the study were 22.7° C in March, 22.7° C in April, 23.3° C in May, and 24.7° C in June. The amount of rainfall during the spray period was relatively low for the study area. Cumulative rainfall values were 5.21 mm in March, 5.91 mm in April, 4.12 mm in May, and 2.3 mm in June.

## Discussion

This study evaluated the efficacy of two bait spray techniques, and quantified the impact of variable sanitation on *B. dorsalis* trapping and fruit infestation data. An additional component of successful IPM approaches applied for the area-wide control of this fly species is the Male Annihilation Treatment (MAT) through use of the male-specific parakairomone lure methyl eugenol (ME). The impact of MAT was not quantified here because ME was used as a way of suppressing male populations and not as a treatment factor. This lure (+ toxicant) has already been used for successful eradication of *B. dorsalis* from Rota (Steiner et al. 1965), Saipan (Steiner et al. 1970), and Okinawa (Koyama et al. 1984). The effectiveness of combining suppression techniques including MAT in an area-wide approach against *B. dorsalis* was demonstrated in the Kamuela area of Hawaii Island during a 6 yr period (Vargas et al. 2010).

The trapping data presented here indicate that GF-120 applied to bait stations performed as well as foliar bait sprays in suppressing *B. dorsalis* from treated plots for the first 10 weeks that followed the first bait spray (i.e. from 19 March to 27 May). This trapping period corresponded with significant decreases in incidence of infestation of 71.4 and 63.1% for plots with bait stations and foliar sprays, respectively, relative to control plots. For the last three weeks of the study (i.e. from 28 May to 17 June) there was a decrease in the effectiveness of the bait sprays as determined by trap captures, and fruit infestation rates were, on average, 54.5 and 45.4% lower for plots with bait stations and foliar sprays, respectively, than control plots. Overall, substantially less GF-120 (∼ 42%) was applied to PLMs than in foliar applications, and this resulted in cost-savings as well as release of less insecticide into the environment.

Use patterns of GF-120 for foliar applications against *B. dorsalis* were evaluated previously by Piñero et al. (2009a) in the same papayagrowing area. These authors reported that GF-120 applied weekly either to all rows (every other tree), or to every 5th row (every tree), in combination with good sanitation successfully reduced both the density of female *B. dorsalis* and levels of fruit infestation. In that study, however, a more conservative bait spray approach that involved applications both to the foliage of papaya trees (using a 10% solution) and to border plants adjacent to treated areas (using a 40% solution) was undertaken owing to the comparatively high populations of *B. dorsalis* present. Under that regime border sprays accounted for about 80% of total GF-120 applied weekly. At the onset of the present study, population densities of female *B. dorsalis* were comparatively low and border areas were not sprayed. Thus, the present study represents a reduced bait application rate compared to that of Piñero et al. (2009a).

Variability in sanitation practices provided an opportunity to estimate the impact of this cultural practice on the numbers of female *B. dorsalis* captured in traps and the incidence of fruit infestation. Previously (Piñero et al. 2009a), it was documented that the numbers of fallen papayas were positively correlated with the numbers of female *B. dorsalis* trapped in control plots, a result that was confirmed in the present study. The nonsignificant differences for trapping and incidence of infestation data between treated and control plots recorded for the last weeks of the study may be explained by a decrease in the number of female *B. dorsalis* captured in control plots during late May and June. This seems to be due to improved sanitation in control plots over time. In contrast, sanitation efforts in some of the treated plots showed no such improvement. The lack of correlation between sanitation data and either trap catches or infestation levels in control plots by the second fruit sampling seems to support this explanation. Thus, the results of this study indicate that application of reduced amounts of GF-120 may not be enough to protect fruit from being infested by *B. dorsalis* in plots with poor sanitation, emphasizing once more the need to practice proper sanitation for successful fruit fly management in papaya orchards.

To qualify as a viable alternative to foliar bait sprays, PLMs should also be cost-competitive. In the present study, the foliar application of GF-120 required an average of 0.25 1 of undiluted GF-120/ha/week, resulting in a total cost of $8.32/ha/week. In contrast, application of ca. 20 ml of a 20% solution of GF-120 to 30 PLMs/ha (the average density used in this study) required an average of 0.12 1 of undiluted GF-120/ha/week, for a total of $4.00/ha/week. The projected cost of spraying GF-120 weekly to papaya foliage using a 10% solution is $432.60/ha/year assuming no reapplication after rainfall events, and $515.80 in the hypothetical (yet conservative) situation that 10 re-applications are needed in one year. In contrast, the projected cost of bait applied to PLMs once a week using a 20% solution is $208.0/ha/year. The cost of materials to make one PLM was around $6.50 (for a total of $195/ha), an amount that can be reduced nearly by half if cheaper materials (e.g. a zip tie or Velcro) instead of shelf brackets are used for attachment to tree trunks or branches of host trees in other agroecosystems. The annual cost of bait and materials needed to make PLMs is $403.0/ha, clearly less than the cost associated with foliar applications. It is also important to consider that foliar sprays require more equipment (e.g. backpack sprayers) and more time for application than PLMs. We believe this comparison demonstrates the economic wisdom of PLMs for this purpose.

A need to develop improved lures and “attract-and-kill” devices including bait stations for successful fruit fly control has already been recognized (IAEA, 2007; Heath et al. 2009). The so-called Papaya Leaf Mimic (PLM), which represents a supernormal visual stimulus of papaya foliage, was developed in Hawaii in response to an imperative need to protect GF-120 against rainfall. Previous behavioral research conducted indicated that PLMs have the potential to be used as an open system to which insecticidal baits can be applied not only due to their rain-fastness properties, but also because the behavioral response of female flies to GF-120 applied onto PLMs is enhanced and the period of bait attractiveness is extended for at least one week (Piñero et al. 2009b). We believe that this visually-attractive bait station also provides a standardized technique for evaluating bait formulations, thus allowing for more precise comparisons over time, among fruit fly species, and across geographical areas.

In conclusion, both spray methods evaluated effectively controlled *B. dorsalis* in papaya orchards under the conditions of this study when performed in combination with proper sanitation. Papaya Leaf Mimics compared favorably to foliar bait sprays despite a substantial reduction (∼ 42%) in the amount of bait applied. Thus, these bait stations represent a simple, efficient, and economical method of delivering insecticidal baits to control fruit flies, and a safer alternative to foliar sprays. Further research should be directed to determine the optimal density of bait stations, the relationship between the need for border sprays and levels of fruit fly pressure, and the possibility of manipulating the habitat to increase the efficacy of this attract-and-kill system.

## Abbreviations

PLM: papaya leaf mimic
